# Differences in Personal Recovery Among Individuals with Severe Mental Disorders in Private and Supported Accommodations: An Exploratory Study

**DOI:** 10.3390/ijerph22081173

**Published:** 2025-07-25

**Authors:** Alessandra Martinelli, Tecla Pozzan, Doriana Cristofalo, Chiara Bonetto, Camilla D’Astore, Elena Procura, Corrado Barbui, Mirella Ruggeri

**Affiliations:** 1 Unit of Epidemiological Psychiatry and Digital Mental Health, IRCCS Istituto Centro San Giovanni di Dio Fatebenefratelli, 25125 Brescia, Italy; 2 Psychiatry Unit, Integrated University Hospital Trust, 37126 Verona, Italy; tecla.pozzan@gmail.com (T.P.); camilla.dastore@aovr.veneto.it (C.D.); corrado.barbui@univr.it (C.B.); mirella.ruggeri2@gmail.com (M.R.); 3 Department of Neurosciences, Biomedicine and Movement Sciences, University of Verona, 37124 Verona, Italy; doriana.cristofalo@univr.it (D.C.); chiara.bonetto@univr.it (C.B.); 4 Mental Health Center, Isola della Scala, Ospedale di Bussolengo, 37051 Verona, Italy; procura.elena@gmail.com; 5 WHO Collaborating Centre for Research and Training in Mental Health and Service Evaluation, Department of Neuroscience, Biomedicine and Movement Science, Section of Psychiatry, University of Verona, 37124 Verona, Italy

**Keywords:** mental health recovery, housing, social support, severe mental disorder, outcome assessments

## Abstract

People with severe mental disorders (SMD) face long-term functional impairments requiring integrated, community-based, recovery-oriented care. Italy provides two main housing models for people with SMD: private accommodation (PA) and supported accommodation (SA). This exploratory study investigated differences in recovery outcomes across these settings using the Mental Health Recovery Star (MHRS). A six-month longitudinal study was conducted within the South Verona Community Mental Health Service. Nineteen trained mental health professionals assessed 25 people with SMD (14 in PA, 11 in SA) at baseline (BL) and follow-up (FU) using standardized tools for recovery (MHRS), functioning, psychopathology, functional autonomy, and needs. Group comparisons and within-group changes were analyzed using paired and independent *t*-tests. At BL, people with SMD in PA showed better functioning (*p* = 0.040) and fewer needs than those in SA (*p* = 0.008). Recovery goals differed, with people with SMD in PA focusing on health and networks, while people with SMD in SA emphasized functioning. At FU, people with SMD in PA improved across all MHRS domains (*p* < 0.001), with significant reductions in symptom severity and unmet needs. People with SMD in SA showed targeted improvements in functioning, autonomy, and MHRS social networks (*p* < 0.001), with increases in met needs but non-significant changes in unmet needs. When comparing PA and SA at FU, the differences were relatively modest. Recovery is achievable in both housing settings, although outcomes differ. People with SMD in PA experienced broader improvements, while people with SMD in SA progressed in their prioritized areas, likely reflecting more complex initial needs. These findings underscore the value of aligning recovery-oriented care with the specific needs and contexts of different residential settings. Further research is needed to confirm and expand these results.

## 1. Introduction

Severe mental disorders (SMD) are long-term conditions characterized by significant impairments in daily functioning, including challenges in work, education, social relationships, and self-care, ultimately limiting full participation in society [1,2]. These disorders encompass a range of mental health conditions such as schizophrenia, bipolar disorder, major depressive disorder, and other disorders [3,4]. Although they represent a relatively small portion of the population—approximately 4% of individuals with mental health conditions—people with SMD often experience a high level of need while receiving low service coverage, a phenomenon described by Killaspy as “low volume, high needs” [5]. Because of their complex and persistent nature, people with SMDs require integrated and comprehensive approaches to care that combine medical treatment with psychosocial and community support [1,6,7].

From the 1970s onward, deinstitutionalization emerged as a transformative movement in mental health care, aiming to reduce reliance on long-term psychiatric hospitalization and promote community-based alternatives [1,2]. This process took different forms across countries. While some systems continue to depend heavily on hospital-based services [3,4,5], many—particularly in Western Europe—have shifted toward models centered on social inclusion, autonomy, and human rights [6,8]. These countries have developed services that enable individuals with long-term mental health conditions to live independently or in supported housing environments, depending on their level of need. Importantly, the presence or absence of psychiatric hospitals alone does not adequately reflect the quality or recovery-orientation of a mental health system; as highlighted by Salisbury et al., a truly deinstitutionalized system is one that provides appropriate care settings based on patients’ needs and promotes autonomy and social integration through comprehensive, rights-based community services, actively addressing the stigma associated with SMD [9,10].

In this context, European mental health policy has increasingly emphasized the personal recovery model—not merely as a clinical approach, but as a multidimensional concept, process, and guiding orientation for mental health systems [11]. Personal recovery refers to a strengths-based framework that supports individuals in leading meaningful lives, even with ongoing symptoms and functional challenges. It emphasizes principles of hope, self-determination, and empowerment through collaborative, person-centered, and evidence-based care, including shared decision-making [12,13,14,15]. Recovery involves recognizing individual strengths and goals, fostering equal partnerships between people with SMD and professionals [12,16], and often includes both progress and setbacks along the way [17,18]. This approach upholds human rights and is linked to better symptoms, functioning, quality of life, satisfaction with care, and reduced service needs [19,20,21,22].

Recovery-oriented care is considered most effective when developed within community-based settings rather than institutional environments, to counteract the risks of marginalization and reinforce human rights and social inclusion [11,23].

Within this model, Community Mental Health Services (CMHSs) are tasked with offering personalized, integrated care that evolves with the service patient’s needs. Evidence supports that recovery-oriented practices within CMHSs have been associated with significant improvements in patients’ self-management, self-efficacy, and autonomy [12,24]. This model has also contributed to better health and social outcomes [21,25,26]. Recovery-oriented practices are increasingly recognized as essential for aligning CMHSs with the evolving needs of contemporary mental health care, and have been positively linked to enhanced treatment effectiveness and reductions in overall healthcare costs [27,28,29].

Italy represents a particularly advanced example in this regard. Following the 1978 mental health reform (Law 180), Italy has fully eliminated psychiatric hospitals, alongside Iceland, and built a comprehensive, community-based mental health care system [9,30] While many countries—such as those in Central and Eastern Europe—still face challenges in realizing deinstitutionalization and rely partly on institutional care [9,31,32], Italy has shifted to a system in which people with SMDs live either in private accommodation (PA) or in supported accommodation (SA) designed according to individualized support needs [33].

Although other countries (e.g., Denmark, the UK, the Netherlands) also offer supported housing, the Italian model is distinctive in its systemic integration of these options into a CMHS network without the use of hospitals [34,35,36]. It operates on a progressive care model, enabling patients to move between more and less intensive settings depending on their level of autonomy and recovery stage. This dynamic model ensures individualized care while promoting long-term goals such as self-management and social integration [37]

This exploratory study aimed to examine personal recovery outcomes among individuals with SMD living in PA and SA within the South Verona CMHS. Both groups participated in the same 6-month recovery-oriented intervention, the Mental Health Recovery Star (MHRS)—a patient-centered tool designed to facilitate personal recovery and to track progress across key domains of personal recovery [5,38,39,40,41,42]. 

The primary objective of the study was to evaluate differences in personal recovery outcomes between the two residential settings after the 6-month intervention. The secondary objective was to assess whether differences were also observed in other relevant dimensions, including psychopathology, functioning, and needs for care.

## 2. Materials and Methods

### 2.1. Study Design

This study was reviewed and approved by the Institutional Review Board (IRB)/Research Ethics Committee of the University Hospital Trust of Verona (reference 34950, dated 30 May 2018). Written informed consent was obtained from all participating people with SMD and mental health professionals.

This small exploratory study was conducted from May 2017 to October 2018 at the South Verona CMHS, as part of a system rooted in evidence-based practices and the bio-psycho-social model of care [43]. CMHS have played a central role in Italy’s mental health system since the process of deinstitutionalization. These services are responsible for providing diagnosis, treatment, and psychosocial support. Organized at the district level, each CMHS serves a catchment area of approximately 100,000 residents, thereby promoting accessibility and continuity of care [44]. A pre-post design was adopted.

Data were collected at recruitment (baseline (BL)) and at the six-month follow-up (FU), aligned with the typical evaluation timeframe used in Italian rehabilitation settings, such as day centers and SA. Given the limited sampling pool, a modest number of participants was anticipated, and the study was therefore designed to be exploratory.

### 2.2. Participants

Participants, both professionals and people with SMD, were selected using purposive sampling based on eligibility and willingness to participate.

Eligible mental health professionals were trained in the MHRS and consented to perform assessments at two time points. They had between one and three individuals with SMD under their care. Of the 45 professionals trained between May and October 2017, 19 fulfilled these inclusion criteria. The remaining 26 were excluded for the following reasons:

(1) Employment outside the South Verona CMHS (n = 15),
(2) Refusal to participate (n = 8),
(3) Inability to recruit a suitable service user (n = 3).

The final sample was predominantly female (78.9%), with medical doctors—primarily psychiatry residents—constituting 36.8% of the group. A substantial majority (73.6%) were employed in multidisciplinary community-based teams. The mean length of professional experience among participants was 137 months (SD = 122.2).

To support consistent MHRS use, professionals attended monthly supervision and educational sessions led by certified trainers. A total of 12 meetings, with a mean of 12 attendees each, covered topics including data collection, recovery-oriented practices, MHRS implementation, motivational interviewing, and shared decision-making. Trainers also offered individual support when needed.

People with SMD were considered eligible based on clinical and functional criteria consistent with international definitions of SMD [3,4]. Specifically, the inclusion criteria were as follows: (1) being under the care of a trained key professional for MHRS at the South Verona CMHS, (2) having a confirmed diagnosis of a severe and persistent mental disorder (such as schizophrenia, bipolar disorder, or other disorder with significant functional impairment), (3) living within the service catchment area, (4) being aged 18–65, and (5) providing informed consent for participation in assessments at two time points. Functional severity and chronicity were assessed by the key professionals, who also verified eligibility. Exclusion criteria were as follows: (1) moderate to severe intellectual disability [45], (2) acute psychiatric hospitalization or severe psychopathological symptoms at the time of BL, with severity assessed using the Health of the Nation Outcome Scales (HoNOS), with exclusion applied if the symptom items scored a 4 (indicating a severe or very severe problem) [46,47]. A total of 25 individuals meeting these criteria were identified and recruited by the key professionals.

### 2.3. Clinical Tools and Their Characteristics

The Verona Department of Mental Health (DMH) database and the South Verona Psychiatric Case Register [48] were used to collect socio-demographic, clinical, and service utilization data for individuals with SMD. The selection of assessment instruments was carried out collaboratively by the research team, certified MHRS trainers, and experienced rehabilitation practitioners to align with established evaluation protocols used in rehabilitation settings within the South Verona CMHS. Following specialized training and under the supervision of the research team, designated professionals conducted standardized assessments at BL and FU.

Developed in 2007 by Triangle Consulting for the Mental Health Providers Forum, the MHRS is designed to facilitate collaborative, “expert-to-expert” relationships between service users and mental health professionals. The tool has gained widespread adoption both in the United Kingdom and internationally. An Italian version was introduced in 2013, and to date, more than 8000 professionals have received training in its use. Although concerns have been raised regarding its inter-rater reliability, the MHRS is widely appreciated for its strong internal consistency and its emphasis on recovery, shared decision-making, and user empowerment.

The MHRS is scored using a 10-point scale representing key life areas enclosed in 4 main domains:

(1) Physical and mental health: managing mental health, self-care, addictive behavior
(2) Activities and functioning: living skills, work, responsibilities
(3) Self-image: identity and self-esteem, trust and hope
(4) Networks: social networks, relationships

Patients and professionals assess progress with the “Scale of Change”, based on the Transtheoretical Model’s five recovery stages [49]—from feeling stuck, passing by, accepting help, believing, and learning to achieving self-reliance. Following the assessment, a collaborative care plan is developed, identifying up to three personalized recovery goals.

In addition to the MHRS, a set of standardized clinical and psychosocial assessment tools was administered to all people with SMD by the staff under the supervision of the research team to enable a multidimensional evaluation of change over time. These included measures of symptom severity, global and social functioning, rehabilitation progress, and perceived needs for care. A visual representation of the Mental Health Recovery Star (MHRS), along with an illustrative case example, is provided in Supplementary Figure S1 to support understanding of the tool’s structure and its use in practice [5,38,39,40,41,42].

The Global Assessment of Functioning (GAF) [50] scale provides a single global rating of psychological, social, and occupational functioning. Scores range from 0 to 100, with higher scores indicating better overall functioning. The scale is divided into 10-point intervals, each anchored by descriptive guidelines to facilitate consistent scoring.

The Health of the Nation Outcome Scales (HoNOS) [46,47] consists of 12 items that assess symptom severity and social functioning. Each item is rated on a scale from 0 (no problem) to 4 (severe problem), yielding a total score ranging from 0 to 48, with higher scores indicating symptom severity and social functioning. This tool offers a concise overview of clinical and psychosocial difficulties.

The Monitoring of the Pathway of Rehabilitation (MPR) [51,52] includes 10 items, each composed of four sub-items. These cover domains such as autonomy, social skills, and work ability. Scoring is domain-specific and utilizes Likert-type or categorical response formats, depending on local adaptation. The MPR is designed to monitor progress in psychiatric rehabilitation and functional recovery over time. Lower scores indicate better functional autonomy.

The Camberwell Assessment of Need (CAN) [53,54] examines 22 areas of need, each rated from both the clinician’s and the patient’s perspectives. Needs are categorized as “no need,” “met need,” or “unmet need,” allowing for a detailed summary of total, met, and unmet needs. This instrument supports individualized care planning by identifying both clinical and social support requirements.

The assessments were administered as follows. The MHRS was completed collaboratively by the service user and the staff member. Professionals completed the HoNOS, GAF, and MPR, while the CAN was administered in two parallel versions—one completed by the service user (CAN-patient) and one by the professional (CAN-staff).

### 2.4. Statistical Analysis

Descriptive data were reported as frequencies, means, and standard deviations. The normality of continuous variables was confirmed using the Kolmogorov–Smirnov test, thus permitting the use of parametric tests. Comparisons between people with SMD living in PA and in SA at BL and at FU were made using *t*-tests for independent samples (continuous characteristics) and Fisher’s exact tests (dichotomous characteristics). Changes between scores on the standardized assessment tools between BL and FU were investigated using the *t*-test for repeated measurements. All tests were bilateral with a significance level set at 0.05. Statistical analyses were performed using the SPSS 22.0 program.

## 3. Results

### 3.1. Mental Health Professionals’ Assessments

A total of 19 mental health professionals conducted the 25 BL assessments, with six professionals evaluating two people with SMD each. As shown in Table A1, which presents the number of evaluations by discipline, rather than individual assessors, medical doctors conducted a higher proportion of evaluations in PA (42.9%), while support workers were the most frequent evaluators in SA (36.4%). The average years of professional experience were higher among evaluators working in SA compared to those in private accommodations, although this difference was not statistically significant (*p* = 0.262).

### 3.2. People with SMD Characteristics at Baseline

As presented in Table 1, 25 people with SMD were included in the study, 14 in PA and 11 in SA. No significant differences were observed between groups in terms of age, marital status, education, employment status, age at first psychiatric contact, clinical diagnosis, years old at first contact with psychiatric service, number of acute ward admissions in a lifetime, number of psychotropic drugs, physical comorbidity (e.g., dyslipidemia, hypothyroidism), substance misuse or gambling problem.

**Figure 1:**
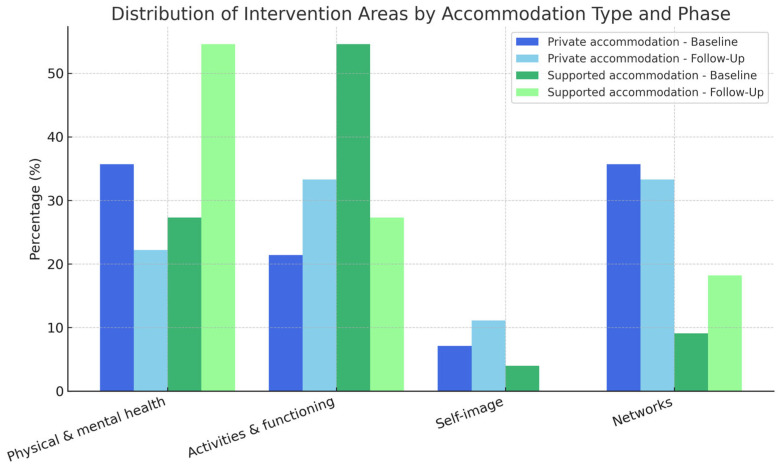
Prioritizing area of the MHRS work plan by accommodation at BL and FU.

People with SMD in PA demonstrated higher mean scores than those in SA in specific MHRS domains, including “Physical and mental health” (*p* = 0.039) and, particularly, “Addictive behavior” (*p* = 0.049). As shown in Table A2 and Figure 1, at BL, people with SMD in PA prioritized goals in “Physical and mental health” and “Networks”, while those in SA prioritized goals in “Activities and functioning”.

In terms of clinical assessments, people with SMD in PA scored significantly higher on the GAF compared to those in SA (*p* = 0.040), indicating better overall functioning.

People with SMD in SA had significantly higher levels of clinical and social needs, as indicated by both the CAN-Patient and CAN-Staff ratings. These included higher total needs (*p* = 0.041 and *p* = 0.008, respectively) and met needs (*p* = 0.021 and *p* = 0.006, respectively).

### 3.3. Clinical and Functional Changes of People with SMD from Baseline to Follow-Up According to Accommodation

Table 2 summarizes changes in clinical and functional outcomes of people with SMD according to accommodation from BL to six-month FU. Although both PA and SA people with SMD showed improvements in romantic relationships (as they prioritized that as a goal at BL) and occupational status, these changes were not statistically significant.

In the PA group, significant improvements were observed in all MHRS domains, alongside a significant increase in the overall MHRS score (*p* < 0.001). Among those in SA, significant improvements were found in the overall MHRS score (*p* < 0.001) and, specifically, in the domains “Activities and functioning” (*p* < 0.001) and “Networks” (*p* = 0.001).

In people with SMD living in PA, symptom severity (HoNOS) decreased significantly (*p* < 0.001) and functional autonomy (MPR) (*p* = 0.041) and functioning (GAF) (*p* < 0.001) improved. In people living in SA, a significant improvement was found only in functional autonomy (MPR) (*p* = 0.005).

Needs assessment (CAN) indicated a significant reduction in total needs and unmet needs for both users (*p* = 0.023 and *p* = 0.003) and staff (*p* = 0.006 and *p* = 0.002) in the PA group. The ratio of met to unmet needs also improved. In the SA group, significant increases were found in met needs from both user (*p* = 0.008) and staff (*p* = 0.015) perspectives, although reductions in unmet needs were not statistically significant.

Changes in recovery goal prioritization across accommodation types are shown in Table A2 and Figure 1. Among users in PA, a statistically significant difference was observed across intervention areas between baseline and follow-up (*p* = 0.011). Although we cannot attribute the significance to a specific category, a descriptive decrease in the proportion of people with SMD prioritizing “Physical and mental health” was noted (from 35.7% to 22.2%). In the SA group, the proportion of people with SMD prioritizing this domain increased from 27.3% to 54.6%, although the overall change across areas was not statistically significant (*p* = 0.176).

When comparing PA and SA at FU, the differences were relatively modest. A statistically significant difference was observed between PA and SA only in “Activities and functioning” (*p* = 0.020) of the four MHRS domains. Similar values of functioning at FU (*p* = 0.218) were observed. Slightly lower scores in psychopathology in PA were observed but they were not statistically significant (*p* = 0.830). A higher functional autonomy in PA was observed but it was not statistically significant (*p* = 0.282). Patient-rated and staff-rated needs showed better ratios of met/unmet needs in PA, with a significant difference for the staff-rated and user-rated total need and total met needs both in SA and PA.

## 4. Discussion

The findings of this study offer an insightful perspective on recovery outcomes among individuals with SMD living in different types of accommodation in Italy. At BL, both groups were broadly similar in terms of socio-demographic and diagnostic characteristics, supporting an initial level of similarity. However, more detailed assessments using standardized tools revealed important differences between the two groups. Specifically, individuals in SA showed lower levels of functioning and a higher number of total needs and unmet needs at BL compared to those in PA. Furthermore, MHRS assessments indicated that people with SMD living in SA began with significantly lower scores in the “Physical and mental health” domain, suggesting a less advanced stage in the recovery process. These findings imply that, although all participants met the criteria for SMD, the level of complexity and severity of their conditions—and potentially the impact of their living environment—differed meaningfully between the two groups. People with SMD in SA were likely experiencing a more severe or complex form of the disorder at study entry.

Interestingly, despite these BL disparities, individuals in PA showed more consistent and widespread improvements across nearly all measured domains from BL to FU. This included significant gains in recovery outcomes, reductions in symptom severity, and improvements in autonomy and overall functioning, as well as notable decreases in both total and unmet needs with an improved ratio of met to unmet needs suggesting a positive shift in perceived care adequacy. Functional improvements, such as social functioning and autonomy, are critical aspects of recovery. Individuals in PA might experience these improvements more rapidly due to fewer constraints and greater initial capacity for independence [55,56].

In contrast, individuals in SA also improved but their gains were more modest and concentrated in specific domains—particularly in areas they had prioritized at BL, including “Activities and functioning”. Reductions in unmet needs were not statistically significant in this group, though met needs did decrease. This pattern suggests that while both groups benefited from recovery-oriented CMHS, individuals in PA may have had more capacity to translate these supports into broader functional improvements.

The greater severity at BL among SA residents likely influenced both their starting point and their rate of recovery over six months. SA residents often start with greater severity of symptoms, which can influence their initial recovery outcomes and rate of improvement. Higher initial severity can slow the rate of recovery, but individuals with severe symptoms can still show substantial improvements over time [57,58]. Furthermore, SA provides essential stability and support, which is crucial for those with higher initial severity, but their progress might be more gradual. It is also important to note that the more modest improvements observed in SA residents do not necessarily reflect a lower recovery potential. Rather, they may indicate that these individuals are already in an environment appropriately matched to their level of need, where progress occurs at a steadier, individualized pace [58,59].

The shift in goal prioritization—from “Physical and mental health” in PA (which became less dominant at FU) to increasing emphasis on this domain in SA—highlights a positive alignment between perceived need and intervention focus, particularly among those starting from a more impaired BL. This alignment is facilitated by multidisciplinary approaches, recovery-oriented practices, and consumer-centered goal setting, ultimately leading to improved health outcomes [36].

Overall, the magnitude of improvement from BL to FU was more marked in PA across several outcomes, yet the FU values in both groups were largely comparable. This suggests that while the starting points differed (e.g., lower baseline functioning in SA), both groups benefited from the intervention. The lack of strong between-group differences at FU reinforces the idea that recovery can occur across different residential settings, albeit perhaps following different outcomes. The fact that SA participants “caught up” in some domains (e.g., functioning) is encouraging, and the smaller gains in some recovery outcomes may reflect a slower or more complex recovery process in these individuals.

From a clinical perspective, these findings highlight the importance of tailoring recovery-oriented interventions to the specific needs and capacities of people with SMD based on their residential context. For individuals in SA, who may begin with more complex needs, steady and sustained progress should be supported through flexible, individualized care planning. These results underscore the value of SA as a stabilizing environment where recovery can unfold at a personalized pace, reinforcing the principle that recovery is not linear and must be defined by the individual’s own goals and context [54,55,56].

### Strengths and Limitations

While this study provides initial insights into potential differences in recovery outcomes between people with SMD living in PA and those in SA, further research is needed to fully understand the observed differences. As a small and exploratory study, these findings should be interpreted with caution. Improvements measured through the MHRS and other outcome tools may reflect natural changes over time rather than the specific impact of the collaborative care planning approach. Additionally, this study was conducted within a single mental health service, limiting the generalizability of the results to other contexts.

Although we provided a detailed description of the standardized assessment tools used—including the MHRS, GAF, HoNOS, MPR, and CAN—the administration of these measures by clinical staff under research supervision may introduce potential biases that were not fully controlled or reflected upon. Factors such as who completed the assessments, and how or where they were completed, could have influenced the results—particularly given differences in carer–user relationships across accommodation types. For example, closer relationships in PA settings might affect ratings. Discrepancies between CAN staff and patient versions across settings could further reflect such bias, though this was not explored in the current analysis.

Another important limitation is the absence of the involvement of people with SMD in the study design, which may have affected the relevance and applicability of the research. The use of multiple statistical tests may reduce the overall power of the study, meaning that the findings should be viewed as preliminary indications rather than definitive conclusions.

While the absence of randomization is understandable given the real-world, service-based context of the intervention, it is a key limitation of the study. Similarly, the lack of a control group comprising institutionalized individuals reflects the unique structure of the Italian mental health system, where long-stay psychiatric hospitals have been replaced entirely by community-based housing such as supported and private accommodations. These two residential models represent the full spectrum of housing options for individuals with SMD in Italy.

## 5. Conclusions

This exploratory study suggests that personal recovery is achievable for individuals with SMD living in both PA and SA settings and following a structured, recovery-oriented intervention. While individuals in PA appeared to show broader improvements across domains, those in SA demonstrated targeted progress in priority areas, potentially reflecting differences in BL functioning and needs. Both groups showed improvements in functioning, symptoms, and needs for care over time, although differences between groups remained. These findings may indicate that recovery-oriented interventions like the MHRS can be flexibly applied to support individuals with varying levels of complexity, though this requires further investigation.

The observed patterns warrant deeper exploration into how residential context and clinical severity interact in shaping recovery outcomes.

Future research with larger samples and extended FU periods is needed to validate these preliminary findings and to better understand how recovery processes unfold across different care settings.

## Figures and Tables

**Table 1:** People with SMD: sociodemographic and clinical characteristics, recovery, functioning, psychopathology and functional autonomy at BL according to accommodation.

	Private Accommodation (N = 14)	Supported Accommodation (N = 11)	Total (N = 25)	* *p*-Value *t* Test or Fisher’s Exact Test
Sociodemographic and clinical characteristics				
Age in years, mean (SD)	40.2 (10.6)	42.3 (9.4)	41.1 (9.9)	0.617
Marital status Single Partnered	10 (71.4%) 4 (28.6%)	9 (81.8%) 2 (18.2%)	19 (76.0%) 6 (24.0%)	0.452
Educational achievement Lower education (primary/middle school only) Higher education (high school/further education)	8 (57.1%) 6 (42.9%)	5 (45.5%) 6 (54.5%)	13 (52.0%) 12 (48.0%)	0.430
Work Employed Unemployed	6 (42.9%) 8 (57.1%)	5 (45.5%) 6 (54.5%)	11 (44.0%) 14 (56.0%)	0.607
Primary clinical diagnosis Schizophrenia spectrum disorders Others	8 (57.1%) 6 (42.9%)	9 (81.8%) 2 (18.2%)	17 (68.0%) 8 (32.0%)	0.190
Years old at first contact with psychiatric service, mean (SD)	25.2 (10.4)	25.1 (6.6)	25.2 (8.8)	0.973
Number of acute ward admissions lifetime, mean (SD)	1.0 (0.0)	1.0 (0.0)	1.0 (0.0)	-
Number of psychotropic drugs, mean (SD)	2.5 (1.2)	3.9 (2.6)	3.1 (2.0)	0.084
Physical comorbidity (e.g., dyslipidemia, hypothyroidism), mean (SD)	0.3 (0.5)	0.4 (0.5)	0.3 (0.5)	0.694
Substance misuse or gambling problem, mean (SD)	0.6 (0.6)	1.1 (0.2)	0.8 (1.1)	0.257
Rating scale assessments				
MHRS, mean (SD) Physical and mental health Managing mental health Self-care Addictive behavior Activities and functioning Living skills Work Responsibilities Self-image Identity and self-esteem Trust and hope Networks Social networks Relationships	6.6 (1.3) 7.2 (1.1) 6.1 (1.7) 7.3 (1.9) 8.1 (2.8) 7.0 (1.8) 6.3 (2.2) 6.1 (4.7) 8.6 (7.3) 6.4 (1.6) 6.3 (1.5) 6.5 (1.9) 5.6 (1.9) 5.6 (1.9) 5.6 (2.4)	5.6 (1.7) 5.8 (2.0) 5.6 (1.9) 6.3 (2.8) 5.5 (3.5) 5.8 (1.7) 5.6 (1.8) 4.7 (2.4) 7.3 (2.6) 5.5 (1.8) 5.5 (2.1) 5.6 (1.6) 5.0 (2.0) 4.9 (2.1) 5.1 (2.8)	6.2 (1.5) 6.6 (1.7) 5.9 (1.8) 6.8 (2.3) 6.9 (3.3) 6.5 (1.8) 5.3 (2.2) 5.5 (2.6) 8.0 (2.2) 6.0 (1.7) 5.9 (1.8) 6.1 (1.8) 5.4 (1.9) 5.3 (2.3) 5.4 (2.6)	0.078 **0.039** 0.485 0.292 **0.049** 0.107 0.367 0.176 0.129 0.199 0.261 0.200 0.416 0.430 0.604
GAF, mean (SD)	63.9 (11.2)	52.1 (16.0)	58.7 (14.5)	**0.040**
HoNOS, mean (SD)	11.6 (4.6)	13.9 (6.5)	12.6 (5.5)	0.303
MPR, mean (SD)	8.9 (1.7)	8.3 (1.4)	8.6 (1.6)	0.328
CAN patient, total needs, mean (SD) Total met needs Total unmet needs Ratio met/unmet needs	8.4 (4.6) 5.8 (3.5) 2.6 (2.7) 2.2	12.1 (3.9) 9.4 (3.6) 2.7 (2.9) 3.5	10.0 (4.6) 7.4 (3.9) 2.6 (2.7) 2.8	**0.041** **0.021** 0.891 -
CAN staff, total needs, mean (SD) Total met needs Total unmet needs Ratio met/unmet needs	8.7 (4.1) 6.0 (3.1) 2.7 (2.9) 2.2	13.4 (3.7) 9.9 (3.3) 3.5 (2.5) 2.8	10.8 (4.5) 7.7 (3.7) 3.0 (2.7) 2.6	**0.008** **0.006** 0.510 -

* p-values in bold denote statistical significance at the *p* < 0.05 level. MHRS—Mental Health Recovery Star—process of recovery. GAF—Global Assessment of Functioning—functioning. HoNOS—Health of the Nation Outcome Scales—psychopathology. MPR—Monitoring of the Pathway of Rehabilitation—functional autonomy. CAN—Camberwell Assessment of Need—needs for care.

**Table 2:** People with SMD: changes in sociodemographic and clinical characteristics, process of recovery, functioning, psychopathology and functional autonomy by accommodation from BL to 6-month FU and between PA and SA.

	BL Private Accommodation (N = 14)	FU Private Accommodation (N = 14)	* *p*-Value Paired *t*-Test	BL Supported Accommodation (N = 11)	FU Supported Accommodation (N = 11)	*p*-Value Paired *t*-Test or Fisher’s Exact Test	*p*-Value Paired *t*-Test or Fisher’s Exact Test PA vs. SA at FU
Sociodemographic and clinical characteristics							
Marital status Single Partnered	10 (71.4%) 4 (28.6%)	8 (57.1%) 6 (42.9%)	1.000	9 (81.8%) 2 (18.2%)	8 (72.7%) 3 (27.3%)	0.055	0.352
Work Employed Unemployed	6 (42.9%) 8 (57.1%)	8 (57.1%) 6 (42.9%)	0.103	5 (45.5%) 6 (54.5%)	6 (54.5%) 5 (45.5%)	0.061	0.393
Number of psychotropic drugs, mean (SD)	2.4 (1.2)	2.1 (1.0)	**<0.001**	4.1 (2.5)	4.2 (2.5)	**<0.001**	**0.035**
Substance misuse or gambling problem, mean (SD)	0.6 (0.6)	0.6 (0.7)	**<0.001**	1.1 (0.2)	0.8 (1.3)	**<0.001**	0.666
Rating scale assessments							
MHRS, mean (SD) Physical and mental health Activities and functioning Self-image Networks	6.6 (1.3) 7.2 (1.1) 7.0 (1.8) 6.4 (1.6) 5.6 (1.9)	7.4 (1.2) 7.5 (1.2) 7.7 (1.6) 7.3 (1.6) 6.6 (2.0)	**<0.001** **0.011** **<0.001** **0.028** **<0.001**	5.6 (1.7) 5.8 (2.0) 5.8 (1.7) 5.5 (1.8) 5.0 (2.0)	5.9 (1.3) 6.3 (1.5) 6.0 (1.3) 5.6 (1.7) 5.3 (1.9)	**<0.001** 0.174 **<0.001** 0.091 **0.001**	0.112 0.206 **0.020** 0.207 0.464
GAF, mean (SD)	66.9 (11.2)	66.5 (13.1)	**<0.001**	52.1 (16.0)	66.8 (10.5)	0.054	0.218
HoNOS, mean (SD)	11.6 (4.6)	8.9 (5.6)	**<0.001**	13.9 (6.5)	10.6 (2.7)	0.629	0.830
MPR, mean (SD)	8.9 (1.7)	10.0 (1.3)	**0.041**	8.3 (1.4)	8.6 (1.5)	**0.005**	0.282
CAN patient, total needs, mean (SD) Total met needs Total unmet needs Ratio met/unmet needs	8.4 (4.6) 5.8 (3.5) 2.6 (2.7) 2.2	6 (3.6) 5 (2.9) 1 (1.8) 5	**0.023** 0.064 **0.003**	12.1 (3.9) 9.4 (3.6) 2.7 (2.9) 3.5	11.8 (3.6) 9.9 (3.9) 1.9 (1.6) 5.2	**0.003** **0.008** 0.306	**0.011** **0.009** 0.581 -
CAN staff, total needs, mean (SD) Total met needs Total unmet needs Ratio met/unmet needs	8.7 (4.1) 6.0 (3.1) 2.7 (2.9) 2.2	6.9 (4.0) 5.8 (2.9) 1.1 (2.0) 5.3	**0.006** **0.051** **0.002**	13.4 (3.7) 9.9 (3.3) 3.5 (2.5) 2.8	12.9 (4.0) 3.7 (1.1) 3.1 (1.8) 1.2	**0.012** **0.015** 0.663	**0.012** **0.033** 0.056 -

* p-values in bold denote statistical significance at the *p* < 0.05 level. MHRS—Mental Health Recovery Star—process of recovery. GAF—Global Assessment of Functioning—functioning. HoNOS—Health of the Nation Outcome Scales—psychopathology. MPR—Monitoring of the Pathway of Rehabilitation—functional autonomy. CAN—Camberwell Assessment of Need—needs for care. PA—Private accommodation. SA—Supported accommodation. BL—baseline. FU—follow-up.

## Data Availability

Requests for original (fully anonymized) participant data may be made to the corresponding author.

## Supplementary Materials

The following supporting information can be downloaded at: https://www.mdpi.com/article/10.3390/ijerph22081173/s1, Figure S1: The Mental Health Recovery Star (MHRS) is a collaborative tool used to assess personal recovery across 10 life domains: managing mental health, self-care, living skills, social networks, work, relationships, addictive behaviour, responsibilities, identity and self-esteem, and trust and hope. Service users rate themselves on a scale of 1 (stuck) to 10 (self-reliant) in each domain through 10 steps from being stuck to full self-reliance.

## Abbreviations

CAN	Camberwell Assessment of Need
CMHS	community mental health service
FPS	Personal and Social Functioning Scale
HoNOS	Health of the Nation Outcome Scale
MHRS	Mental Health Recovery Star
MPR	Monitoring of the Path of Rehabilitation

## Appendix A

**Table A1:** Background Characteristics of Mental Health Professionals Involved in Assessments by Accommodation Type.

	Private Accommodation	Supported Accommodation	*p*-Value *t* Test or Fisher’s Exact Test
Gender, male	4 (28.6%)	1 (9.1%)	0.245
Discipline Medical doctor Support worker Nurse/basic carer Psychologist	6 (42.9%) 4 (28.6%) 2 (14.3%) 2 (14.3%)	3 (27.3%) 4 (36.4%) 2 (18.2%) 2 (18.2%)	0.885
Months working in mental health, mean (SD)	112.4 (105.7)	168.6 (139.1)	0.262

**Table A2:** Prioritizing area of the MHRS work plan by accommodation at BL and FU.

	BL Private Accommodation (N = 14)	FU Private Accommodation (N = 9)	*p*-Value Fisher’s Exact Test	BL Supported Accommodation (N = 11)	FU Supported Accommodation (N = 11)	*p*-Value Fisher’s Exact Test
Area/s of intervention						
Physical and mental health	5 (35.7%)	2 (22.2%)	0.011	3 (27.3%)	5 (54.6%)	0.176
Activities and functioning	3 (21.4%)	3 (33.3%)		6 (54.6%)	3 (27.3%)	
Self-image	1 (7.1%)	1 (11.1%)		1 (4.0%)	0 (0.0%)	
Networks	5 (35.7%)	3 (33.3%)		1 (9.1%)	2 (18.2%)	

## References

[1] Reed G.M. (2024). What’s in a name? Mental disorders, mental health conditions and psychosocial disability. World Psychiatry.

[2] WHO Europe (2022). The WHO European Framework for Action to Achieve the Highest Attainable Standard of Health for Persons with Disabilities 2022–2030.

[3] WHO (2017). Helping People with Severe Mental Disorders Live Longer and Healthier Lives.

[4] Parabiaghi A., Bonetto C., Ruggeri M., Lasalvia A., Leese M. (2006). Severe and persistent mental illness: A useful definition for prioritizing community-based mental health service interventions. Soc. Psychiatry Psychiatr. Epidemiol..

[5] Killaspy H. (2014). The ongoing need for local services for people with complex mental health problems. Psychiatr. Bull..

[6] European Council of the European Union (2024). Disability in the EU: Facts and Figures.

[7] Caldas de Almeida J.M., Killaspy H. (2011). Long-Term Mental Health Care for People with Severe Mental Disorders. Report for the European Commission. https://health.ec.europa.eu/document/download/53cb0f32-1746-4a25-ad0e-228a68b37fe5_en.

[8] United Nations (2006). UN Convention on the Rights of Persons with Disabilities. United Nations General Assembly A/61/611. https://www.un.org/disabilities/documents/convention/convoptprot-e.pdf.

[9] Taylor Salisbury T., Killaspy H., King M. (2016). An international comparison of the deinstitutionalisation of mental health care: Development and findings of the Mental Health Services Deinstitutionalisation Measure (MENDit). BMC Psychiatry.

[10] Martinelli A. (2025). The key pillars of psychosocial disability: A European perspective on challenges and solutions. Front. Psychiatry.

[11] NICE (2020). Rehabilitation for Adults with Complex Psychosis.

[12] Slade M., Amering M., Farkas M., Hamilton B., O’HAgan M., Panther G., Perkins R., Shepherd G., Tse S., Whitley R. (2014). Uses and abuses of recovery: Implementing recovery-oriented practices in mental health systems. World Psychiatry.

[13] SLAM/SWLSTG (2010). Recovery is for All: Hope, Agency and Opportunity in Psychiatry.

[14] Rapp C.A., Goscha R.J. (2012). The Strengths Model A Recovery-Oriented Approach to Mental Health Services.

[15] Beale V., Lambric T. (1995). The Recovery Concept: Implementation in the Mental Health System.

[16] Farkas M., Gagne C., Anthony W., Chamberlin J. (2005). Implementing recovery oriented evidence based programs: Identifying the critical dimensions. Community Ment. Health J..

[17] Deegan P.E. (1996). Recovery as a journey of the heart. Psychiatr. Rehabil. J..

[18] Leamy M., Bird V., Le Boutillier C., Williams J., Slade M. (2011). A conceptual framework for personal recovery in mental health: Systematic review and narrative synthesis. Br. J. Psychiatry.

[19] Killaspy H., Harvey C., Brasier C., Brophy L., Ennals P., Fletcher J., Hamilton B. (2022). Community-based social interventions for people with severe mental illness: A systematic review and narrative synthesis of recent evidence. World Psychiatry.

[20] Martinelli A., Ruggeri M. (2020). The impact on psychiatric rehabilitation of recovery oriented-practices. J. Psychopathol..

[21] Liberman R.P. Recovery from Disability: Manual of Psychiatric Rehabilitation. https://catalog.nlm.nih.gov/permalink/01NLM_INST/1o1phhn/alma9913063143406676.

[22] WHO—Regional Office for Europe (2015). The European Mental Health Action Plan 2013–2020.

[23] WHO—European Ministerial Conference on Mental Health (2005). Mental Health Declaration for Europe “Facing the Challenges, Building Solution”. https://iris.who.int/handle/10665/326566.

[24] Mccabe R., Whittington R., Cramond L., Perkins E. (2018). Contested understandings of recovery in mental health. J. Ment. Health.

[25] Carozza P. (2006). Principi di Riabilitazione Psichiatrica: Per un Sistema di Servizi Orientato alla Guarigione.

[26] Barnes T.R.E., Pant A. (2005). Long-term course and outcome of schizophrenia. Psychiatry.

[27] Frank H., Kalidindi S., Killaspy H., Glenn R. (2016). Enabling Recovery: The Principles and Practice of Rehabilitation Psychiatry.

[28] Bee P., Owen P., Baker J., Lovell K. (2015). Systematic synthesis of barriers and facilitators to service user-led care planning. Br. J. Psychiatry.

[29] Needham C., Mary Q., Carr S. Co-Production: An Emerging Evidence Base for Adult Social Care Transformation. https://lx.iriss.org.uk/sites/default/files/resources/briefing31.pdf.

[30] Thornicroft G., Tansella M. (2013). The balanced care model for global mentalhealth. Psychol. Med..

[31] WHO (2021). Mental Health ATLAS 2020.

[32] WHO (2022). World Mental Health Report: Transforming Mental Health for All.

[33] Martinelli A., Dal Corso E., Pozzan T., Cristofalo D., Bonetto C., Ruggeri M. (2023). Addressing Challenges in Residential Facilities: Promoting Human Rights and Recovery While Pursuing Functional Autonomy. Psychiatr. Res. Clin. Pract..

[34] Martinelli A., Iozzino L., Ruggeri M., Marston L., Killaspy H. (2019). Mental health supported accommodation services in England and in Italy: A comparison. Soc. Psychiatry Psychiatr. Epidemiol..

[35] van Veldhuizen R., Delespaul P., Kroon H., Mulder N. (2015). FlexibleACT & Resource-group ACT: Different Working Procedures Which Can Supplement and Strengthen Each Other. A Response. Clin. Pract. Epidemiol. Ment. Health.

[36] Munch Nielsen C., Hjorthøj C., Helbo A., Madsen B.P., Nordentoft M., Baandrup L. (2025). Effectiveness of a multidisciplinary outreach intervention for individuals with severe mental illness in supported accommodation. Nord. J. Psychiatry.

[37] Martinelli A., Iozzino L., Pozzan T., Cristofalo D., Bonetto C., Ruggeri M. (2022). Performance and effectiveness of step progressive care pathways within mental health supported accommodation services in Italy. Soc. Psychiatry Psychiatr. Epidemiol..

[38] Placentino A., Lucchi F., Scarsato G., Fazzari G., Gruppo rex.it (2017). La Mental Health Recovery Star: Caratteristiche e studio di validazione della versione italiana. Riv. Psichiatr..

[39] Lloyd C., Williams P.L., Machingura T., Tse S. (2015). A focus on recovery: Using the Mental Health Recovery Star as an outcome measure. Adv. Ment. Health.

[40] Dickens G., Weleminsky J., Onifade Y., Surgarman P. (2012). Recovery Star: Validating user recovery. Psychiatrist.

[41] Keen E.L. (2011). The Outcome Star: A Tool for Recovery Orientated Services. Exploring the Use of the Outcome Star in a Recovery Orientated Mental Health Service.

[42] Onifade Y. (2011). The mental health recovery star. Ment. Health Soc. Incl..

[43] Tansella M., Amaddeo F., Burti L., Lasalvia A., Ruggeri M. (2006). Evaluating a community-based mental health service focusing on severe mental illness. The Verona experience. Acta Psychiatr. Scand..

[44] Senato della Repubblica Legislatura XVII (2017). Disegno di Legge. Disposizioni in Materia di Tutela della Salute Mentale Volte All’attuazione e allo Sviluppo dei Princìpi di cui alla Legge 13 Maggio 1978, n. 180. 2850 Italia. https://www.senato.it/export/ddl/full/48103?leg=17.

[45] American Psychiatric Association (2013). Diagnostic and Statistical Manual of Mental Disorders: DSM-5*^TM^*.

[46] Lora A., Bai G., Bianchi S., Bolongaro G., Civenti G., Erlicher A., Maresca G., Monzani E., Panetta B., Von Morgen D. (2001). The italian version of HoNOS (Health of the Nation Outcome Scales), a scale for evaluating the outcome and the severity in mental health services. Epidemiol. Psichiatr. Soc..

[47] Erlicher A., Tansella M. (2012). Health of the Nation Outcome Scales HoNOS: Una Scala per la Valutazione della Gravità e dell’esito nei Servizi di Salute Mentale.

[48] Amaddeo F. (2018). Using large current databases to analyze mental health services. Epidemiol. Prev..

[49] Prochaska J.O., DiClemente C.C. (1986). The Transtheoretical Approach: Crossing Traditional Boundaries of Therapy.

[50] American Psychiatric Association (2000). Diagnostic and Statistical Manual of Mental Disorders, Fourth Edition: DSM-IV-TR.

[51] Martinelli A., Dal Corso E., Pozzan T. (2024). Monitoring of the pathway of rehabilitation (MPR). J. Psychopathol..

[52] Martinelli A., Pozzan T., Corso E.D., Procura E., D’Astore C., Cristofalo D., Ruggeri M., Bonetto C. (2022). Proprietà psicometriche della Scheda di Monitoraggio del Percorso Riabilitativo (MPR). Riv Psichiatr..

[53] Slade M., Loftus L., Thornicroft G. (1999). The Camberwell Assessment of Need (CAN).

[54] Ruggeri M., Lasalvia A., Nicolaou S., Tansella M. (1999). The Italian version of the Camberwell assessment of need (CAN), an interview for the identification of needs of care. Epidemiol. Psichiatr. Soc..

[55] Grammenos S. Comparability of Statistical Data on Persons with Disabilities Across the EU. https://www.europarl.europa.eu/RegData/etudes/STUD/2024/754219/IPOL_STU(2024)754219_EN.pdf.

[56] Barnes S., Carson J., Gournay K. (2022). Enhanced supported living for people with severe and persistent mental health problems: A qualitative investigation. Health Soc. Care Community.

[57] Killaspy H., Priebe S., McPherson P., Zenasni Z., Greenberg L., McCrone P., Dowling S., Harrison I., Krotofil J., Dalton-Locke C. (2019). Predictors of moving on from mental health supported accommodation in England: National cohort study. Br. J. Psychiatry.

[58] Killaspy H., Priebe S., McPherson P., Zenasni Z., McCrone P., Dowling S., Harrison I., Krotofil J., Dalton-Locke C., McGranahan R. (2019). Feasibility randomised trial comparing two forms of mental health supported accommodation (Supported Housing and Floating Outreach); a component of the QUEST (Quality and Effectiveness of Supported Tenancies) study. Front. Psychiatry.

[59] Van Eck R.M., Jelsma A., Blondeel J., Burger T.J., Vellinga A., de Koning M.B., Schirmbeck F., Kikkert M., Boyette L.-L., de Haan L. (2025). The Association Between Change in Symptom Severity and Personal Recovery in Patients with Severe Mental Illness. J. Nerv. Ment. Dis..

