# Supplementary material for: Differences in Personal Recovery Among Individuals with Severe Mental Disorders in Private and Supported Accommodations: An Exploratory Study
Source: Int J Environ Res Public Health. 2025 Jul 25;22(8):1173. doi: 10.3390/ijerph22081173 (PMC12386056; doi:10.3390/ijerph22081173)

**Supplementary Figure 1.** The Mental Health Recovery Star (MHRS) is a collaborative tool used to assess personal recovery across 10 life domains: managing mental health, self-care, living skills, social networks, work, relationships, addictive behaviour, responsibilities, identity and self-esteem, and trust and hope. Service users rate themselves on a scale of 1 (stuck) to 10 (self-reliant) in each domain through 10 steps from being stuck to full self-reliance.

1. Stuck: step 1 and 2
2. Accepting help: step 3 and 4
3. Believing: step 5 and 6
4. Learning: step 7 and 8
5. Self-reliance: step 9 and 10

**Illustrative Case Example (Maria, Age 42)**
Maria is a woman with a diagnosis of bipolar disorder who recently moved into supported accommodation. At baseline, she scored low in *social networks* (2), *work* (3), and *identity and self-esteem* (2), indicating challenges in community connection and confidence. At follow-up, after six months of participation in the recovery-oriented intervention, she showed marked improvement in *managing mental health* (from 4 to 7), *work* (from 3 to 6), and *identity and self-esteem* (from 2 to 6). These changes reflected her increased engagement in vocational training and her growing sense of autonomy and purpose.

**T0 - baseline**

**T1 – folow-up after 6 months of recovery-oriented intervention**

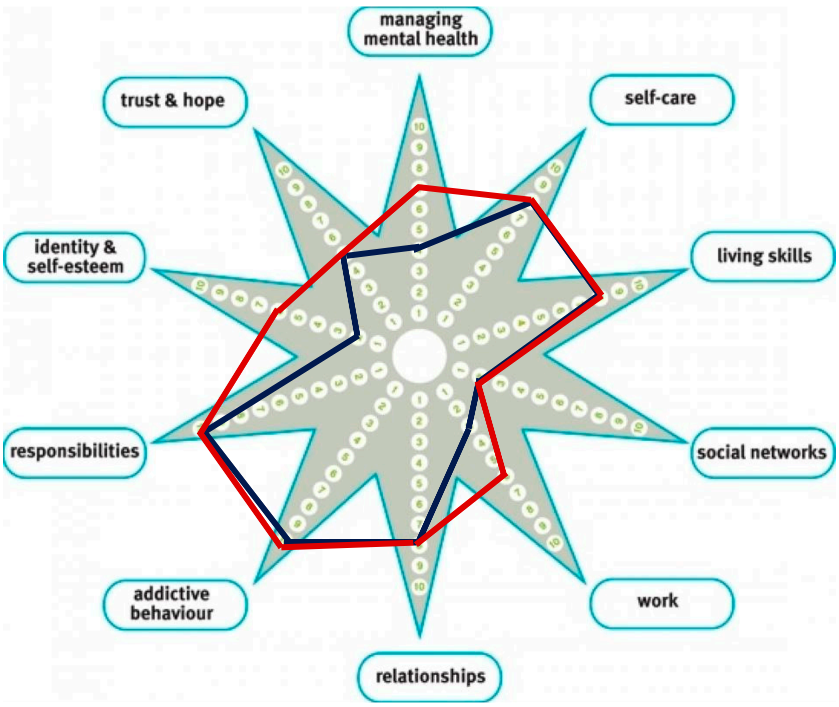

Supplement: Supplementary file 1 [file ijerph-22-01173-s001.zip › ijerph-3709708-supplementary.pdf]
